# Predicting Individual Survival after Curative Esophagectomy for Squamous Cell Carcinoma of Esophageal

**DOI:** 10.1155/2021/5595718

**Published:** 2021-04-03

**Authors:** Zhiyong Zhao, Xiaolong Huang, Ting Gu, Zhu Chen, Limin Gan, Biao Zhu, Ning Wu

**Affiliations:** ^1^Intensive Care Unit, Fudan University Shanghai Cancer Center, Shanghai, China; ^2^Intensive Care Unit, The First Affiliated Hospital of Xiamen University, Xiamen, China; ^3^Zoe Softe Corp, Ltd., Xiamen, China; ^4^Department of Cardio-Thoracic Surgery, Huashan Hospital of Fudan University, Shanghai, China

## Abstract

**Background:**

Esophageal cancer is one of the leading causes of cancer-related death worldwide. Despite the significant progress in the overall treatment of esophageal cancer in recent years, the prognosis for patients who require surgery remains poor.

**Methods:**

The present study investigated the clinicopathological features of 503 patients who underwent radical esophagectomy at Huashan Hospital of Fudan University between January 2005 and January 2015. Nomograms that predicted the esophageal squamous cell carcinoma (ESCC) survival rates were established using the Cox proportional hazard regression model. Discrimination and calibration, which were calculated after bootstrapping, were used as a measure of accuracy.

**Results:**

Multivariate analyses were used to select five independent prognostic variables and build the nomogram. These variables were pathological T stage, pathological N factor, rate of positive LNs, history of chronic obstructive pulmonary disease (COPD) and postoperative sepsis. The nomogram was built to predict the rates for overall survival (OS) and disease-free survival (DFS). The concordance index for the nomogram prediction for OS and DFS was 0.720 and 0.707, respectively. Compared to the conventional TNM staging system, the nomogram had better predictive accuracy for survival (OS 0.720 vs. 0.672, *P* < 0.001; DFS 0.707 vs. 0.667; *P* < 0.001).

**Conclusions:**

The present study incorporated pathological T stage, pathological N factor, rate of positive LNs, history of COPD, and postoperative sepsis into a nomogram to predict the OS and DFS of ESCC patients. This practical system may help clinicians in both decision-making and clinical study design. The assessment of lung function for patients with COPD preoperative, and the control of disease progression are needed. Furthermore, the postoperative infection of patients should be controlled. Further studies may help to extend the validation of this method and improve the model through parameter optimization.

## 1. Introduction

Esophageal cancer is one of the leading causes of cancer-related death worldwide [1]. Despite the significant progress in the overall treatment of esophageal cancer in recent years, the prognosis for patients who require surgery remains poor. The establishment of an accurate cancer staging system would be valuable for both the provision of information and in guiding patient follow-up and subsequent treatments. The most commonly used staging system for esophageal squamous cell carcinoma (ESCC) is the tumor node metastasis (TNM) classification system from the 7^th^ edition of the American Joint Committee on Cancer (AJCC). However, studies have demonstrated that other clinicopathological factors, such as lymph node ratio [2–4], comorbidities [5, 6], and postoperative complications [2], are also significant prognostic variables. Furthermore, there are no models that can concurrently take comorbidities and postoperative complications into account in constructing an accurate predictive model. Hence, the present study is aimed at assessing the comorbidities and postoperative complications in patients with esophageal cancer and designing a nomogram for the prediction of long-term survival in patients with resected ESCC. To the best of the knowledge of the authors, the present study is the first to attempt to establish an ESCC nomogram based on comorbidities and postoperative complications using a relatively large cohort of patients.

## 2. Materials and Methods

A total of 503 patients participated in the present study. These patients underwent potential curative esophagectomy for squamous cell carcinoma of the esophagus between January 2005 and January 2015 in Huashan Hospital at Fudan University, which is a tertiary referral center with significant experience in esophageal surgery. The patients in the present study (1) underwent transthoracic esophagectomy with mediastinal and two-field abdominal lymphadenectomy with R0 resection, (2) had no in-hospital mortality, and (3) did not have other malignancies or distant metastases. The surgical methods used have been previously described [7].

The collected patient information included the demographic information such as age, gender, body mass index (BMI), tobacco use, alcohol use, preoperative albumin, preoperative platelet, preoperative white blood cell (WBC), and preoperative neutrophil to lymphocyte ratio (NLR). Additional variables included comorbidities, clinicopathological features, postoperative complications, and survival.

The comorbidities were identified during the preoperative evaluation of the physician or other healthcare professional notes and subsequently confirmed via appropriate medical tests. These comorbidities included history of cardiovascular disease (previous myocardial infarction, heart failure, peripheral arterial disease, or cerebrovascular disease), history of chronic obstructive pulmonary disease (COPD) [6], history of hepatitis, history of hypertension, and history of diabetes (with or without complications). Renal comorbidities were too rare to include in the statistical analyses.

The clinicopathological factors were evaluated in accordance to the guidelines for clinical and pathological studies on carcinoma of the esophagus. The tumor staging was based on the TNM classification specified by the International Union Against Cancer [8], and depth of invasion and lymph node metastasis were determined based on from the pathology of the surgically resected specimens. The postoperative pathological T (pT), N (pN), and Stage (pStage) factors were used for all cases. For patients who received preoperative therapy, the depth of invasion was determined through both the microscopic distribution of viable cancers, and the scar tissue and disappearance of normal structures, such as the lamina propria and proper muscular layer.

The 7^th^ edition of the AJCC recommends removing a sufficient number of LNs during the operation, and the detection of at least 12 nodes. However, in clinical practice, due to various factors such as individual physical condition, operating conditions, and pathological diagnosis, it remains difficult to ensure the removal of a sufficient number of LNs from each patient. Hence, this may result in the stage migration phenomenon. The metastatic lymph node ratio is the ratio of metastatic LNs to the number of total detected LNs, which may be affected by variability during detection. This variable was included in the present study.

The present study evaluated the postoperative complications that developed within 30 days after esophagectomy, which required either medication or surgical intervention. A postoperative pulmonary complication was defined as the presence of one or more of the following postoperative conditions: initial ventilator support for more than 48 hours or reintubation for respiratory failure, the need for tracheostomy, pneumonia, or acute respiratory distress syndrome (ARDS). Postoperative anastomotic leakage was defined in terms of the clinical signs of leaking, such as erythema, skin edema, emission of pus from a surgical wound or cervical drain, or a radiographically apparent leak confirmed by performing an esophagography or computed tomography, or both. Cardiovascular morbidity was defined as the presence of any cardiac disease or cerebrovascular disease, such as arrhythmia, ischemic heart disease, or pericardial fluid collection, which required pharmacological, electrical, or interventional treatment, or the presence of any thrombosis in line with the common terminology criteria for adverse events (CTCAE) version 4.03 [2]. Sepsis was defined as clinical signs of SIRS along with a culture or visually identified infection.

### 2.1. Statistical Analysis

The statistical analyses were performed using the statistical package R for Windows (version 3.4.2, http://www.r-project.org/). For the purpose of developing the nomograms, the outcome predictor was developed with the clinical experience of the authors, as well as through the search of prior literature. Quantitative data were expressed in median and interquartile range (IQR), and categorical data were expressed numerically and in percentage. The Kaplan-Meier method was used to estimate the OS and DFS. Cox regression analysis was used for the univariate and multivariate analyses. Variables with a *P* value of <0.05 in the univariate analysis were subjected to the multivariable Cox regression analysis. A final model selection was performed using backward stepwise regression with Akaike's Information Criterion (AIC) [9]. Furthermore, the graphical assessment of proportional hazards assumptions and the test of nonlinear terms for significance using analysis of variance (ANOVA) were performed. A nomogram was formulated based on the results of the multivariate analysis using the rms statistical package [10].

Discrimination and calibration were used to test the accuracy of the nomograms. The discrimination of the nomogram was measured using a concordance index (C-index) and the bootstrap bias-corrected estimates of the C-index. Calibration curves, which measure the relationship between the outcomes predicted by the models and the observed outcomes in the patients, were used to assess calibration accuracy in predicting the probability of the overall survival probability and progression-free survival probability for 1, 3, and 5 years. These analyses were performed using a bootstrapping strategy with 200 replications. The nomogram and pathological staging systems were compared using the rcorrp.cens package.

The total points for each patient were calculated according to the established nomogram. Three groups of patients with high, moderate, and low risk of survival were delineated using maximally selected rank statistics, as implemented in the Maxstat package [11]. The survival curves were drawn using the Kaplan-Meier method. Finally, with the risk group as a factor, these were compared using log-rank test.

All statistical tests were two-sided, and *P* values of <0.05 were considered statistically significant.

## 3. Results

### 3.1. Clinicopathologic Characteristics of Patients

A total of 503 patients were enrolled in the present study. The patient characteristics are presented in Table 1. The median age of diagnosis was 62 years old. The median number of resected LNs was 13 (range: 8-19). The majority of patients were male (81.7%). The most common comorbidity was a history of hypertension (34.4%), and a total of 148 (29.4%) patients suffered from postoperative pulmonary complications.

### 3.2. OS and DFS of Patients

The median OS was four years (95% CI: 3.50-4.83 years), and the 1-, 3-, and 5-year OS rates were 82.5%, 57.5%, and 42.3%, respectively. The median DFS was 3.33 years (95% CI: 2.92-4.00 years), and the 1-, 3-, and 5-year disease free rate was 77.6%, 52.1%, and 40.9%, respectively. The median follow-up time was 4.62 years (range: 1.21-17.08 years).

### 3.3. Independent Prognostic Factors

In order to determine the factors that are independently prognostic of patient survival, the OS and DFS were analyzed using the Cox proportional hazards model. Tables 2 and 3 highlight all parameters identified to be of potential significance in the univariate analysis, and these were included in the multivariate analysis. The multivariate analyses indicated that history of COPD, pathological T stage, pathological N factor, rate of positive LNs, and postoperative sepsis were independent risk factors for OS and DFS.

### 3.4. Prognostic Nomogram for OS and DFS

**Figure 1:**
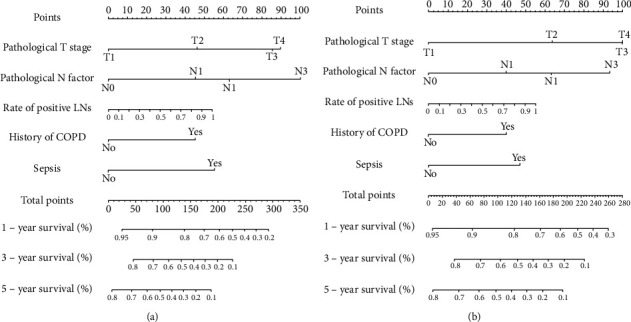
Survival nomogram for patients with resected esophageal squamous cell carcinoma (to use the nomogram, an individual patient's value is located on each variable axis, and a line is drawn upward to determine the number of points received for each variable value. The sum of these number is located on total point axis, and a line is drawn downward to the survival axes to determine the likelihood of 1-, 3- or 5-year survival. (a) is for overall survival; (b) is for disease free survival).

**Figure 2:**
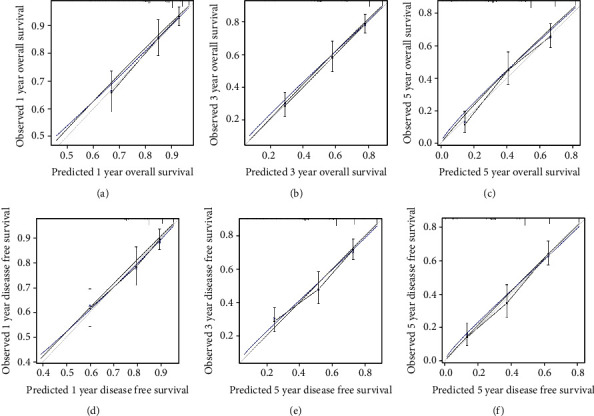
Calibration curve for predicting patient survival at (a, d) 1 year, (b, e) 3 years and (c, f) 5 years in the validation cohort. Nomogram-predicted overall survival/disease free survival (DFS) is plotted on the *x*-axis; observed overall survival/disease free survival is plotted on the *y*-axis.

The prognostic nomograms that integrated all independent factors for OS and DFS in the primary cohort are shown in Figures 1(a) and 1(b), respectively. The calibration plot for the probability of survival at 1/3/5 year(s) after surgery demonstrate the optimal concordance between the nomogram prediction and actual observation (Figure 2).

### 3.5. Validation of Predictive Accuracy of the Nomogram for OS and DFS

The C-index of the nomogram for OS was 0.720 (95% CI: 0.682-0.758), and the bias-corrected C-index was 0.712. The C-index and bias-corrected C-index of the nomogram for DFS were 0.707 (95% CI: 0.670-0.744) and 0.700, respectively. For the pathological stage, the C-index and bias-corrected C-index for OS (0.672 and 0.669, respectively) and DFS (0.669 and 0.666, respectively) were significantly lower than the C-index of the nomogram (*P* < 0.001, *P* < 0.001).

**Figure 3:**
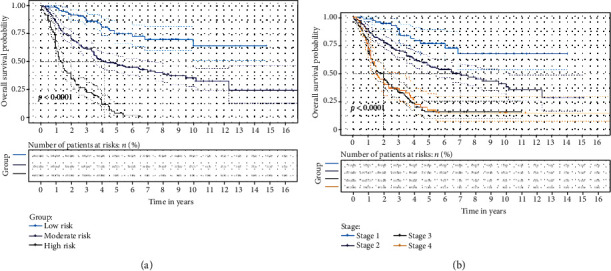
Kaplan-Meier survival curves of the primary cohort categorized by different staging systems for overall survival ((a) established model; (b) American Joint Committee on cancer (AJCC) seventh edition).

**Figure 4:**
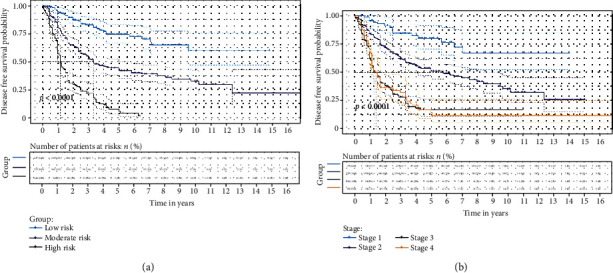
Kaplan-Meier survival curves of the primary cohort categorized by different staging systems for disease free survival ((a) established model; (b) American Joint Committee on cancer (AJCC) seventh edition).

The risk stratification based on the score obtained from the nomogram supported the predictive efficacy in the long-term survival of the established model (Figures 3 and 4). The patients were divided into three risk groups according to their total score for OS (low-risk group: >22 and ≤74, moderate-risk group: >74 and ≤155, and high-risk group: >155 and ≤271) and DFS (low-risk group: >22 and ≤83, moderate-risk group: >83 and ≤161, and high-risk group: >161 and ≤274), respectively.

## 4. Discussion

The present study investigated the predictive factors for long-term survival in the 503 patients who underwent resection of ESCC. The cancer characteristics were closely correlated with the long-term survival of ESCC patients. However, a large number of studies have reported that many other clinicopathological factors are also associated with the prognosis. The present study found that a history of COPD and postoperative sepsis were significantly correlated to OS and DFS in patients with ESCC. A clinical nomogram was developed which included the pathological T stage, pathological N factor, rate of positive LNs, history of COPD, and postoperative sepsis. Subsequently, a risk stratification system was constructed based on the nomogram score. These developed nomograms are more accurate than the conventional staging system for predicting prognosis in ESCC patients, and calibration plots indicated a concordance between prediction and actual observation. The C-index value for OS and DFS was 0.720 and 0.707, respectively.

A number of prior studies have demonstrated that comorbidities have an impact on the prognosis of ESCC patients [5, 6, 12, 13]. A history of COPD is one of the most common conditions, accounting for 11.5% of newly diagnosed ESCC cancer patients. Furthermore, this has an association with significantly worse prognosis [14–16]. COPD is a disease characterized by completely irreversible and usually progressive obstruction of the airways and is associated with inflammation [17]. Furthermore, in patients with ESCC, following esophageal carcinoma resection and intrathoracic gastroesophagostomy, part of the thoracic cavity is occupied by the stomach that has been pulled up. This leads to further impairment of respiratory motion and poor pulmonary function. Second, immune dysfunction plays an important role in the occurrence of COPD [17], which may facilitate the rapid development of microscopic residual disease into clinically manifested recurrence. Third, COPD was found to be a risk factor for pulmonary complications following surgery [18]. Postoperative pulmonary complications may be correlated with worse prognosis [2], although this was not found in the present study. Overall, COPD may play an important role in predicting long-term survival, and the present study revealed that this is an independent predictor of death among patients with ESCC. However, further mechanistic studies are necessary.

N staging is essentially based on the number of metastatic LNs, but the main source of error in the number of metastatic LNs lies in the variation of the total number of examined LNs. In the present study, the median number of examined LNs was 13 (range: 8-19), which can easily result in the stage migration phenomenon in these patients. Furthermore, the present study indicated that present AJCC staging is unable to satisfactorily distinguish between the prognosis for stage III and stage IV groups. These results are demonstrated in Figures 3(b) and 4(b). The ratio of metastatic lymph nodes is affected by the number of examined LNs. In addition, the present study found that the lymph node ratio is an independent predictor of survival for patients undergoing esophagectomy for ESCC, which is consistent with prior literatures [3, 4, 19, 20]. The lymph node ratio may compensate for the deficiencies in the AJCC nodal categories. Hence, combining the lymph node ratio and AJCC nodal categories may more accurately predict the survival, when compared to the present staging system [21].

Sepsis was the only postoperative variable associated with long-term mortality, and this finding is consistent with a previous literature [22]. For cancer patients, the occurrence of postoperative sepsis is associated with aggressive immunosuppression [23], which is potentially associated with cancer recurrence and mortality [21, 22].

The present study has some limitations. First, the present single-center study had a retrospective design. Nonetheless, the study utilized a database of more than 500 cases from a single institution that used relatively standardized surgical techniques and postoperative management, thereby avoiding some of the limitations of multicenter, population-based, or nationwide studies. Second, the present study did not include external validation. Although 200 bootstrap resamples were carried out for internal validation, there is still a risk of bias. Third, patients had a median number of only 13 examined LNs. Thus, this data may not be suitable for patients with more extensive lymphadenectomy. However, previous studies have suggested that extensive lymphadenectomy do not provide any survival benefit. Furthermore, extensive lymphadenectomy introduces additional risks for complications, and may delay postoperative recovery time and reduce quality of life.

## 5. Conclusions

The present study has incorporated pathological T stage, pathological N factor, rate of positive LNs, history of COPD, and postoperative sepsis into a nomogram to predict the OS and DFS of ESCC patients. This practical system may help clinicians in both decision-making and clinical study design. The preoperative assessment of lung function in patients with COPD, and the control disease progression are needed. Furthermore, the postoperative infection of patients should be controlled. Further studies may help to extend the validation of the method, and improve the model through parameter optimization.

## Figures and Tables

**Table 1:** Clinical, epidemiological, and pathological feature.

	Median/*N*	IQR/percentage
Age, year	62	56-67
Sex		
Male	411	81.7%
Female	92	18.3%
BMI, kg/m^2^	22.23	20.07-24.19
Tobacco use		
No	338	67.2%
Yes	165	32.8%
Alcohol use		
No	381	75.7%
Yes	122	24.3%
Comorbidities		
History of hypertension		
No	330	65.6%
Yes	173	34.4%
History of diabetes		
No	381	75.7%
Yes	122	24.3%
History of COPD		
No	445	88.5%
Yes	58	11.5%
History of hepatitis		
No	428	85.1%
Yes	75	14.9%
History of cardiovascular disease		
No	422	83.9%
Yes	81	16.1%
Preoperative albumin, g/L	41.00	39.00-43.00
Preoperative platelet, ^∗^10^9^	194.00	157.00-239.00
Preoperative WBC, ^∗^10^9^	5.88	4.89-7.23
Preoperative NLR	2.25	1.67-3.17
Length of tumor, cm	3.00	2.00-4.50
Location of tumor		
Upper	82	16.3%
Middle	302	60.0%
Lower	119	23.7%
Differentiation of tumor		
Well	65	12.9%
Moderate	299	59.4%
Poor	139	27.6%
Pathological T stage		
T1	73	14.5%
T2	146	29.0%
T3	242	48.1%
T4	42	8.3%
Pathological N factor		
N0	263	52.3%
N1	140	27.8%
N2	70	13.9%
N3	30	6.0%
Pathological stage		
I	72	14.3%
II	227	45.1%
III	135	26.8%
IV	69	13.7%
Number of dissected LNs	13	8-19
Number of positive LNs	0	0-2
Rate of positive LNs	0	0-0.17
Postoperative complications		
Postoperative pulmonary complications	148	29.4%
Postoperative anastomotic leakage	29	5.8%
Postoperative cardiovascular disease	107	21.3%
Sepsis	34	6.8%

Data are expressed as median (interquartile range, IQR), *N* (percentage, %). BMI: body mass index; COPD: chronic obstructive pulmonary disease; NLR: neutrophil to lymphocyte ratio.

**Table 2:** Univariable and multivariable Cox analysis of prognostic factors for overall survival in 503 patients with esophageal squamous cell carcinoma.

	Univariable analysis		Multivariable analysis	
	HR (95% CI)	*P* value	HR (95% CI)	*P* value
Age, year	1.01 (0.99-1.03)	0.11		
Sex				
Male	Ref			
Female	0.74 (0.54-1.02)	0.07		
BMI, kg/m^2^	0.97 (0.93-1.01)	0.12		
Tobacco use				
No	Ref			
Yes	1.11 (0.86-1.42)	0.42		
Alcohol use				
No	Ref			
Yes	1.14 (0.87-1.49)	0.35		
Comorbidities history of hypertension				
No	Ref			
Yes	1.10 (0.85-1.41)	0.48		
History of diabetes				
No	Ref			
Yes	1.18 (0.90-1.56)	0.23		
History of COPD				
No	Ref		Ref	
Yes	1.50 (1.08-2.10)	0.02	1.79 (1.27-2.53)	<0.001
History of hepatitis				
No	Ref			
Yes	1.03 (0.74-1.44)	0.84		
History of cardiovascular disease				
No	Ref			
Yes	0.92 (0.66-1.29)	0.62		
Preoperative albumin, g/L	0.96 (0.93-0.99)	0.03		
Preoperative platelet, ∗10^9^	1 (0.998-1.002)	0.63		
Preoperative WBC, ^∗^10^9^	1.06 (1.004-1.129)	0.04		
Preoperative NLR	1.06 (1.006-1.117)	0.03		
Length of tumor, cm	1.18 (1.11-1.25)	<0.001		
Location of tumor				
Upper	Ref			
Middle	0.86 (0.62-1.19)	0.35		
Lower	0.91 (0.62-1.33)	0.62		
Differentiation of tumor				
Well	Ref			
Moderate	1.69 (1.09-2.62)	0.02		
Poor	2.19 (1.37-3.49)	<0.001		
Pathological T stage				
T1	Ref		Ref	
T2	2.51 (1.43-4.41)	0.001	1.82 (1.02-3.22)	0.004
T3	4.76 (2.80-8.10)	<0.001	3.01 (1.74-5.20)	<0.001
T4	6.33 (3.44-11.67)	<0.001	3.17 (1.66-6.07)	<0.001
Pathological N factor				
N0	Ref		Ref	
N1	2.24 (1.67-3.00)	<0.001	1.80 (1.30-2.49)	<0.001
N2	3.78 (2.34-6.10)	<0.001	2.26 (1.27-4.00)	0.005
N3	5.73 (4.10-8.01)	<0.001	3.63 (2.32-5.68)	<0.001
Pathological stage				
I	Ref			
II	2.43 (1.41-4.18)	0.001		
III	6.82 (3.95-11.77)	<0.001		
IV	6.61 (3.71-11.76)	<0.001		
Number of dissected LNs	1.01 (0.99-1.02)	0.37		
Number of positive LNs	1.13 (1.10-1.17)	<0.001		
Rate of positive LNs				
Postoperative complications	10.06 (6.34-15.97)	<0.001	2.01 (0.91-4.43)	0.008
Postoperative pulmonary complications	1.20 (0.92-1.55)	0.18		
Postoperative anastomotic leakage	1.59 (0.97-2.60)	0.07		
Postoperative cardiovascular disease	1.10 (0.82-1.46)	0.54		
Sepsis	1.82 (1.18-2.82)	0.007	2.04 (1.31-3.18)	0.002

BMI: body mass index; COPD: chronic obstructive pulmonary disease; NLR: neutrophil to lymphocyte ratio.

**Table 3:** Univariable and multivariable Cox analysis of prognostic factors for disease free survival in 503 patients with esophageal squamous cell carcinoma.

	Univariable analysis		Multivariable analysis	
	HR (95% CI)	*P* value	HR (95% CI)	*P* value
Age, year	1.01 (0.99-1.02)	0.17		
Sex				
Male	Ref			
Female	0.75 (0.54-1.02)	0.07		
BMI, kg/m^2^	0.97 (0.94-1.01)	0.20		
Tobacco use				
No	Ref			
Yes	1.07 (0.84-1.36)	0.60		
Alcohol use				
No	Ref			
Yes	1.12 (0.86-1.45)	0.42		
Comorbidities				
History of hypertension				
No				
Yes	1.11 (0.87-1.41)	0.42		
History of diabetes				
No	Ref			
Yes	1.26 (0.96-1.64)	0.09		
History of COPD				
No	Ref		Ref	
Yes	1.42 (1.02-1.97)	0.04	1.65 (1.17-2.31)	0.004
History of hepatitis				
No	Ref			
Yes	0.96 (0.69-1.33)			
History of cardiovascular disease				
No	Ref			
Yes	0.91 (0.66-1.26)			
Preoperative albumin	0.96 (0.93-0.99)	0.04		
Preoperative platelet, ^∗^10^9^	1.001 (0.999-1.003)	0.436		
Preoperative WBC, ^∗^10^9^	1.05 (0.99-1.12)	0.08		
Preoperative NLR	1.05 (0.99-1.11)	0.05		
Length of tumor, cm	1.17 (1.11-1.25)	<0.001		
Location of tumor				
Upper	Ref			
Middle	0.85 (0.62-1.17)	0.33		
Lower	0.93 (0.65-1.35)	0.71		
Differentiation of tumor				
Well	Ref			
Moderate	1.62 (1.07-2.46)	0.02		
Poor	2.04 (1.31-3.18)	0.002		
Pathological T stage				
T1	Ref		Ref	
T2	2.92 (1.67-5.09)	<0.001	2.21 (1.25-3.89)	0.006
T3	5.23 (3.08-8.89)	<0.001	3.46 (2.00-5.97)	<0.001
T4	6.42 (3.48-11.82)	<0.001	3.47 (1.82-6.60)	<0.001
Pathological N factor				
N0	Ref		Ref	
N1	2.09 (1.58-2.78)	<0.001	1.65 (1.30-2.49)	0.002
N2	3.72 (2.37-5.84)	<0.001	2.20 (1.27-3.79)	0.005
N3	5.11 (3.68-7.09)	<0.001	3.20 (2.07-4.93)	<0.001
Pathological stage				
I	Ref			
II	2.80 (1.63-4.81)	<0.001		
III	7.23 (4.19-12.46)	<0.001		
IV	7.02 (3.96-12.45)	<0.001		
Number of dissected LNs	1.01 (0.99-1.02)	0.28		
Number of positive LNs	1.13 (1.10-1.17)	<0.001		
Rate of positive LNs	9.19 (5.8-14.58)	<0.001	1.99 (0.90-4.36)	0.009
Postoperative complications				
Postoperative pulmonary complications	1.21 (0.94-1.56)	0.14		
Postoperative anastomotic leakage	1.44 (0.88-2.35)	0.15		
Postoperative cardiovascular disease	1.11 (0.84-1.47)	0.46		
Sepsis	1.66 (1.07-2.57)	0.02	1.79 (1.16-2.79)	0.009

BMI: body mass index; COPD: chronic obstructive pulmonary disease; NLR: neutrophil to lymphocyte ratio.

## Data Availability

The datasets used and/or analyzed during the current study are available from the corresponding author on reasonable request.

## Abbreviations

ESCC: Esophageal squamous cell carcinoma
COPD: Chronic obstructive pulmonary disease
OS: Overall survival
DFS: Disease-free survival
TNM: Tumor node metastasis
AJCC: American Joint Committee on Cancer
BMI: Body mass index
WBC: White blood cell
NLR: Neutrophil to lymphocyte ratio
ARDS: Acute respiratory distress syndrome
CTCAE: Common terminology criteria for adverse events
IQR: Interquartile range
AIC: Akaike's Information Criterion
ANOVA: Analysis of variance.
